# Lipome géant cervico-dorsal avec lourdeur et attitude vicieuse du rachis cervical: à propos d’un cas à Bamako (Mali)

**DOI:** 10.11604/pamj.2016.25.148.10715

**Published:** 2016-11-11

**Authors:** Youssouf Fofana, Mahamadou Mallé

**Affiliations:** 1 Service de Dermatologie, Centre National d’Appui à la Lutte contre la Maladie, Bamako, Mali; 2 Service de Radiologie, Hôpital Régional de Gao, Mali

**Keywords:** Lipome, géant, cervico-dorsal, Lipoma, giant, cervical-dorsal

## Image en médecine

Les lipomes sont des tumeurs bénignes qui se développent au dépend des adipocytes, le plus souvent encapsulés et de croissance lente. Ils surviennent surtout à l’âge adulte, sans prédilection de race, ni de sexe, avec dans certaines études, une légère prédominance féminine. Le lipome est dit géant lorsque la pièce d’exérèse est supérieure à cinq centimètres. Les lipomes de localisation cervico-dorsale sont rares. Nous rapportons le cas d'un patient âgé de 40 ans, présentant une tuméfaction cervico-dorsale évoluant depuis 2008 et augmentant progressivement de volume. Au centre de la tumeur se trouvait un placard achromique post ulcéreux entouré par de rayures pigmentées. Depuis quelques temps, le patient signalait une sensation de lourdeur et une attitude vicieuse du rachis cervical. Nous avions évoqué trois hypothèses diagnostiques, un lipome le plus probable, un liposarcome, et une tumeur royale. Le scanner sans injection du produit de contraste a conclu à un lipome. L’excision chirurgicale de la tumeur a permis d’extraire la graisse bien encapsulée. Le lipome pesait 1025 grammes et mesurait 17x15x6 cm. L’histologie de la pièce opératoire a confirmé le résultat du Scanner. Les suites opératoires étaient simples avec disparition de la lourdeur cervicale.

**Figure 1:**
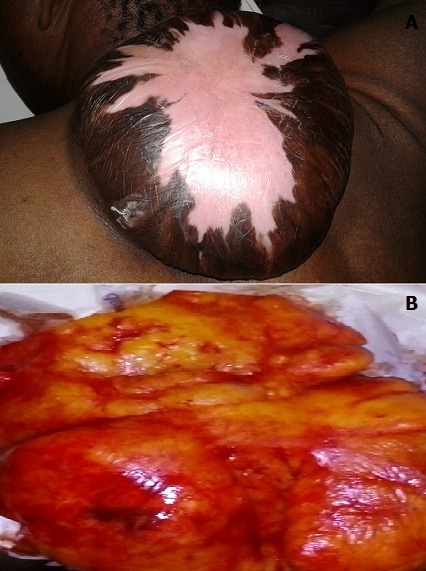
A) volumineuse masse cervico-dorsale de consistance molle chez un patient de 40 ans faisant évoquer le diagnostic de lipome; B) masse graisseuse bien encapsulée après excision de la tumeur

